# A Comparison between High-Performance Countercurrent Chromatography and Fast-Centrifugal Partition Chromatography for a One-Step Isolation of Flavonoids from Peanut Hulls Supported by a Conductor like Screening Model for Real Solvents

**DOI:** 10.3390/molecules28135111

**Published:** 2023-06-29

**Authors:** Mats Kiene, Svenja Blum, Gerold Jerz, Peter Winterhalter

**Affiliations:** Institute of Food Chemistry, Technische Universität Braunschweig, Schleinitzstrasse 20, 38106 Brauschweig, Germany; m.kiene@tu-braunschweig.de (M.K.); g.jerz@tu-braunschweig.de (G.J.)

**Keywords:** peanut hull, *Arachis hypogaea*, eriodictyol, luteolin, high-performance countercurrent chromatography, fast-centrifugal partition chromatography, semi-preparative isolation, ESI-MS-MS, 1D/2D-NMR, conductor like screening model for real solvents, *COSMO-RS*

## Abstract

Peanut hulls (*Arachis hypogaea*, *Leguminosae*), which are a side stream of global peanut processing, are rich in bioactive flavonoids such as luteolin, eriodictyol, and 5,7-dihydroxychromone. This study aimed to isolate these flavonoid derivatives by liquid-liquid chromatography with as few steps as possible. To this end, luteolin, eriodictyol and 5,7-dihydroxychromone were isolated from peanut hulls using two different techniques, high-performance countercurrent chromatography (HPCCC) and fast-centrifugal partition chromatography (FCPC). The suitability of the biphasic solvent system composed of *n*-hexane/ethyl acetate/methanol/water (1.0/1.0/1.0/1.5; *v*/*v*/*v*/*v*) was determined by the *Conductor like Screening Model for Real Solvents* (*COSMO-RS*), which allowed the partition ratio *K_D_*-values of the three main flavonoids to be calculated. After a one-step HPCCC separation of ~1000 mg of an ethanolic peanut hull extract, 15 mg of luteolin and 8 mg of eriodictyol were isolated with purities over 96%. Furthermore, 3 mg of 5,7-dihydroxychromone could be isolated after purification by semi-preparative reversed-phase liquid chromatography (semi-prep. HPLC) in purity of over 99%. The compounds were identified by electrospray ionization mass spectrometry (ESI-MS) and nuclear magnetic resonance spectroscopy (NMR).

## 1. Introduction

### 1.1. Ingredients and Antioxidative Effects of Peanut Hulls

**Figure 1:**
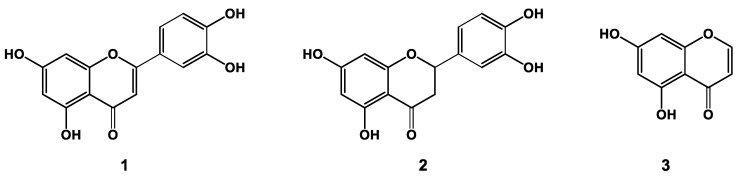
Chemical structures of luteolin (**1**) and eriodictyol (**2**), and 5,7-dihydroxychromone (**3**).

*Arachis hypogaea* (*Leguminosae*) is an annual herb whose geocarpic kernels are used as raw products or after roasting in various processed food products, such as snacks and cereals. The global harvest volume of peanuts in 2021/2022 was approximately 50.3 million tons [1]. Today’s larger cultivation regions include *inter alia* China, India, and Egypt. The yellow wooden pericarp peanut hulls are removed before being processed into products such as peanut oil and butter or roasted peanut kernels. The contents of hull material range between 230–300 g/kg of recovered peanuts [2], resulting in 10–13 million tons annually available as a valuable side stream for upcycling into innovative products. Peanut hull (or shell) extracts contain a high concentration of natural antioxidants [3], such as flavonoids revealing potential in animal models for the treatment of Alzheimer’s disease and diabetes mellitus [4,5,6,7]. Positive neurotrophic functionalities were also reported for ethanolic peanut hull extracts [8], whereby the principal bioactive recoverable metabolites include luteolin (**1**), eriodictyol (**2**), and 5,7-dihydroxychromone (**3**) (cf. Figure 1). Compounds **1** and **2** show effects against Alzheimer’s disease [5,7] and diabetes mellitus [4,6], so their isolation is of interest for possible use in food supplements.

The flavonoids **1**, **2** and the polyphenol derivate **3** undergo concentration changes during maturation. Predominantly, eriodictyol is found in the hull of immature peanuts, whereas luteolin content is increased in mature peanut hulls. The composition of the flavonoids changed from 50% eriodictyol (**2**), 41% luteolin (**1**), and 9% 5,7-dihydroxychromone (**3**) to 38% **2** and 52% **1**, whereby the content of **3** remained unchanged [9].

### 1.2. Liquid-Liquid Chromatography Separation Techniques

Previously, a three-step isolation protocol combined silica gel adsorption and size exclusion column chromatography (Sephadex LH-20), as well as semi-preparative reversed-phase liquid chromatography to recover eriodictyol (**1**) and luteolin (**2**) from peanut hulls [10]. Niu and coworkers described a high-speed counter-current chromatography method for the isolation of luteolin (**1**) and 5,7-dihydroxychromone (**3**) from a peanut hull crude extract [11]. In our study, high-performance countercurrent chromatography (HPCCC) and fast-centrifugal partition chromatography (FCPC) were selected as semi-preparative all-liquid separation techniques that offer predictable parameter settings for a potential process scale-up [12,13]. An additional advantage of these separation techniques is that the irreversible adsorption of target compounds onto the separation system can be avoided due to the absence of a solid stationary phase [14].

The separation principle of liquid-liquid chromatography is based on the differences in the specific partition ratio value (*K_D_*) of compounds between two immiscible phase layers of a solvent system used as mobile- and stationary phases [14]. HPCCC and FCPC have proven to be suitable instruments for the preparative scale isolation of natural compounds from various plant sources and food materials [15,16,17,18]. Countercurrent chromatography can be divided into the *hydrostatic* and *hydrodynamic* principles, FCPC and HPCCC. In the hydrostatic set-up, the stationary phase is retained by an induced centrifugal field in the large set of serially connected separation channels of the FCPC rotor column system due to the high speed (>1000 rpm) rotation. The mobile phase is pumped through the stationary phase in ascending or descending mode, depending on the mode selected [19]. The HPCCC, as a hydrodynamic all-liquid system, implement a double-rotation planetary motion of two or three self-balancing rotating coil systems equipped with wounded multi-layer Teflon tubing (PTFE tube) as support for the chromatographic phases. HPCCC was designed for higher rotational speeds (up to 1600 rpm), enabling higher mobile flow rates and optimized separation times while the resolution is not severely compromised [20,21,22].

### 1.3. COSMO-RS Supported Solvent System Selection

Evaluating an appropriate two-phase solvent system for a specific separation problem can be time-consuming in the CCC workflow. Therefore, in-silico-based models for testing a wider range of such solvent systems can be implemented in a short time without requiring laboratory work with chemicals and necessary infrastructure. Here, the *Conductor-like Screening Model for Real Solvents* (*COSMO-RS*) was applied to calculate the liquid-liquid equilibrium (LLE) and solutes partition ratio (*K_D_*) [23,24]. *COSMO-RS* only requires the molecular structure of the solute molecules and the specific solvent compositional values in the two-phase layer. The intermolecular forces of solvents in a liquid system can be described as pairwise interactions of surface segments. In the software tool, this leads to a highly efficient and rather fast calculation routine of the chemical potentials of any compound in a two-phase solvent system [25]. Previous work with *COSMO-RS* in the context of partition chromatography demonstrated the suitability of this in-silico method for calculating both LLE and *K_D_*-values [26,27]. Calculated LLEs were shown to be comparable to experimentally determined values by gas chromatography employing a thermal conductivity detector. For the separation of standard mixtures, the approximation was successfully achieved by comparing in-silico *K_D_*-values from *COSMO-RS* with the results of classical shake flask solvent system evaluation tests [14]. Likewise, predicted *K_D_*-values for target compounds in a planned HPCCC or FCPC experiment could be precalculated with *COSMO-RS* [27,28,29,30]. Applications for *COSMO-RS* as a solvent selection tool in all-liquid chromatography have been described for the separation of flavone and isoflavone glycosides from *Sophora japonica* fruits [31] and for the simultaneous isolation of cannabidiol (CBD) and removal of pesticides removal from hemp extracts (*Cannabis sativa*) [32].

This study aimed to compare two all-liquid chromatography techniques—HPCCC and FCPC—for isolating bioactive compounds from a crude ethanolic extract of peanut hull for upcycling this valuable side stream into innovative products. Furthermore, the in-silico software *COSMO-RS* was implemented for the calculation of LLE of ten two-phase systems and the *partition ratios* (*K_D_*) of luteolin (**1**), eriodictyol (**2**) and 5,7-dihydroxychromone (**3**) in the most promising solvent systems. The study will demonstrate that *COSMO-RS* can be used to find suitable solvent systems to isolate substances from crude extracts in one step using liquid-liquid chromatographic techniques. The chromatographic parameters of both techniques—HPCCC and FCPC—were compared step-by-step. In addition, the ability of *COSMO-RS* to calculate *K_D_*-values of the three flavonoids from the peanut hull extract was evaluated and compared to the results of the experimental shake flask tests.

## 2. Results and Discussion

### 2.1. Extraction and Pre-Analysis of Polyphenols in Extract of Roasted Peanut Hulls

**Figure 2:**
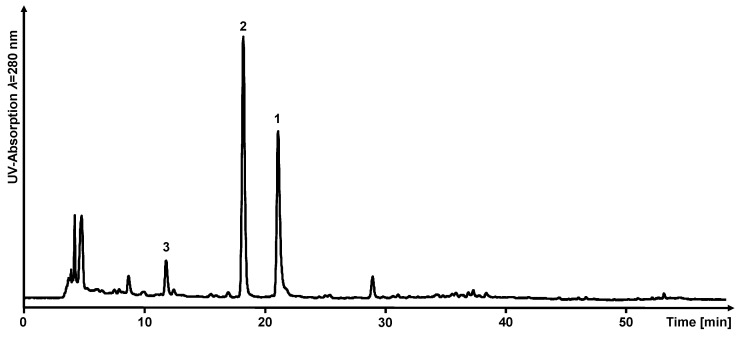
HPLC-UV chromatogram of the peanut hull extract at *λ* 280 nm. (**3**), 5,7-dihydroxychromone *t_R_* 12.4 min; (**2**), eriodictyol *t_R_* 19.2 min; (**1**), luteolin *t_R_* 21.9 min.

In the isolation study, the three target compounds luteolin (**1**), eriodictyol (**2**) and 5,7-dihydroxychromone (**3**) were evaluated to test the suitability for isolation of pure compounds from peanut hull extract based on calculated *K_D_*-values by *COSMOtherm* using two different semi-preparative fractionation techniques (HPCCC and FCPC). To prepare the extract, 58 g of roasted peanut hulls were ground and macerated with an ethanol-water solvent mixture (80/20; *v*/*v*) to yield 4 g of crude polyphenolic extract (7% by weight). By a liquid chromatography-mass spectrometry (LC-ESI-MS) analysis, luteolin (**1**) ([M − H]^−^, *m*/*z* 285) and eriodictyol (**2**) ([M − H]^−^, *m*/*z* 287) were identified as the two major compounds of the extract. Furthermore, the previously reported 5,7-dihydroxychromone (**3**) was directly identified in the extract by LC-ESI-MS/MS with [M − H]^−^ at *m*/*z* 177) [9]. C_18_-HPLC analysis (*λ* 280 nm) revealed a longer retention time for luteolin (**1**) (*t_R_*: 21.9 min) than for eriodictyol (**2**) (*t_R_*: 19.2 min) and 5,7-dihydroxychromone (**3**) (*t_R_*: 12.4 min) (cf. Figure 2).

### 2.2. Evaluation and Prediction of Compound Specific Partition Ratio K_D_-Values by HPLC-UV Analysis and COSMO-RS Calculation of Roasted Peanut Hull Extract

The solvent system was chosen based on preliminary studies and published work on the separation of flavonoids with HSCCC [15]. For the separation of luteolin (**1**) by CCC, no previous data was available. Therefore the suitability for separation of the mixture of principal components, luteolin (**1**), eriodictyol (**2**) and 5,7-dihydroxychromone (**3**), had to be tested. The solvent system *n*-hexane/ethyl acetate/methanol/water (1.0/1.0/1.0/1.5; *v*/*v*/*v*/*v*) used by Du and coworkers [15] and related systems with slightly differing polarity (cf. Table 1) were pre-evaluated by *COSMO-RS* software.

In the first step, the phase equilibria for the solvent systems 1–10 were calculated by *COSMO-RS* using the liquid-extraction module. In the second step, the compound-specific *K_D_*-values were calculated (cf. Table 2). The *K_D_*-values of the target compounds in liquid-liquid countercurrent chromatography should be in the range of 0.4 ≤ *K_D_* ≤ 2.5 [33]. Only the *K_D_*-values for luteolin (**1**), eriodictyol (**2**), and 5,7-dihydroxychromone (**3**) of systems 4–6 were in the postulated range. A *K_D_*-value of 0.30 was calculated for luteolin in system 4, and the *K_D_*-values of eriodictyol (**2**) and 5,7-dihydroxychromone (**3**) in the same system 4 were 0.47 and 0.75, respectively. The *K_D_*-values for the three target compounds in systems 5 and 6 were in the correct range [system 5: **1** (0.75), **2** (1.08), **3** (1.18); system 6: **1** (1.44), **2** (1.96), **3** (1.61)]. Comparison of separation factors [*α*] revealed that system 4 presented the highest separation factors for the compound pairs luteolin/eriodictyol (1.57) and also for eriodictyol/5,7-dihydroxychromone (1.60).

The final selection for solvent system 4 was made by comparison of *COSMO-RS* calculated *K_D_*-values with the predicted values from the shake flask test (cf. Table 2). The shake flask test is an established method for determining the compound-specific *K_D_*-values in the respective biphasic solvent system [14]. Predicted *K_D_*-values were determined by HPLC-UV analysis of the respective phase layers from shake flask experiments with the peanut hull extract. Calculating the HPLC-UV peak area values (A) of the target compounds in the phase layers resulted in the determination of the specific *K_D_*-values. Since the HPCCC separation was in the *head-to-tail* mode and the FCPC separation in the so-called *descending mode*, the following equation (peak area A *upper phase*/peak area A *lower phase*) was used to predict the compound-specific *K_D_*-values (cf. Supplementary Material, Table S2). The predicted *K_D_*-values for the two major compounds, luteolin (**1**) and eriodictyol (**2**) were 0.58 and 0.86, respectively. For 5,7-dihydroxychromone (**3**), a value of 1.56 was determined (cf. Table 2).

The in-silico calculated *K_D_*-values for all three flavonoid derivatives were approximately two-fold lower than those the shake flask experiments predicted. Similar to the shake flask experiments, the predicted separation factors [*α*] of luteolin/eriodictyol and eriodictyol/5,7-dihydroxy-chromone were larger than 1.5. Overall, the calculated *K_D_*-values by *COSMO-RS* reflect the same trends as the predicted *K_D_*-values performed by shake flask experiments. Based on our *COSMO-RS* calculations, solvent system 4 appeared not only suitable for the separation of eriodictyol (**2**) and 5,7-dihydroxychromone (**3**), as previously shown by Du et al. [15] but also luteolin (**1**). For this reason, the solvent system *n*-hexane/ethyl acetate/methanol/water (1.0/1.0/1.0/1.5; *v*/*v*/*v*/*v*) (solvent system 4) was used in the further work.

### 2.3. Isolation of Luteolin, Eriodictyol and 5,7-Dihydroxychromone from Roasted Peanut Hulls by Semi-Preparative HPCCC/FCPC Fractionation and HPLC-Purification

**Figure 3:**
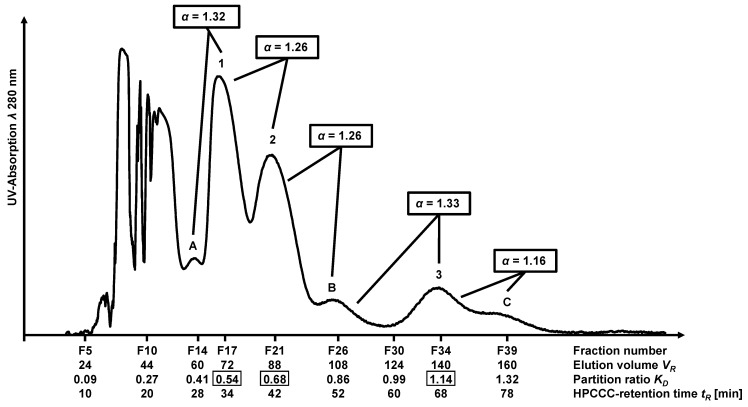
UV chromatogram of the peanut hull extracts HPCCC separation at *λ* 280 nm. (**1**), luteolin (*K_D_* = 0.54); (**2**), eriodictyol (*K_D_* = 0.68); (**3**), 5,7-dihydroxychromone (*K_D_* = 1.14); (**A**), fractions 13–15 (*K_D_* = 0.41); (**B**), fractions 24–29 (*K_D_* = 0.86); (**C**), fractions 37–42 (*K_D_* = 1.32).

Two semi-preparative separations with the same solvent system *n*-hexane/ethyl acetate/methanol/water (1.0/1.0/1.0/1.5; *v*/*v*/*v*/*v*) were performed (cf. Section 2.2), each using 1000 mg of ethanolic peanut hull extract but with two different semi-preparative fractionation techniques (HPCCC and FCPC). The HPCCC and FCPC fractions were screened by thin-layer chromatography (TLC), and fractions were pooled based on the different colors of the compound spots (cf. Supplementary Material, Figures S1 and S2). For the separation by HPCCC, the *head-to-tail* modus was used, yielding a total of 68 fractions in *elution*-mode. The peak labels A (fractions 13–15, *K_D_* = 0.41), B (fractions 24–29, *K_D_* = 0.86), and C (fractions 37–42, *K_D_* = 1.32) in Figure 3 described the peaks next to flavonoid-derivate peaks which were required for the calculation of separation factor *α*. HPCCC separation factors were discussed in detail in Section 2.5.

As shown in Figure 3, luteolin (**1**) eluted at smaller retention volumes compared to eriodictyol (**2**) and 5,7-dihydroxychromone (**3**) in the HPCCC separation. At this stage, pure luteolin (15 mg) and eriodictyol (8 mg) with purities of 96% and 98%, respectively, were obtained using this one-step HPCCC separation method (HPLC-UV analysis *λ* 280 nm). The final purification step of combined HPCCC fractions 32–36 (*K_D_* = 1.14; cf. Figure 3) was realized on an RP-18 semi-preparative column using an isocratic method yielding pure 5,7-dihydroxychromone (**3**) (3 mg) with approximately 99% purity. The LC-ESI-MS and NMR spectral data of the three isolated compounds were listed in Supplementary Material, Tables S3–S6.

This HPCCC method is characterized by the fact that luteolin (**1**) and eriodictyol (**2**) are obtained in very high purities by fast one-step isolation within 80 min. The isolated amounts of luteolin (1.5%) and eriodictyol (0.8%) offer good potential for upscaling due to the reproducibility of the one-step process.

**Figure 4:**
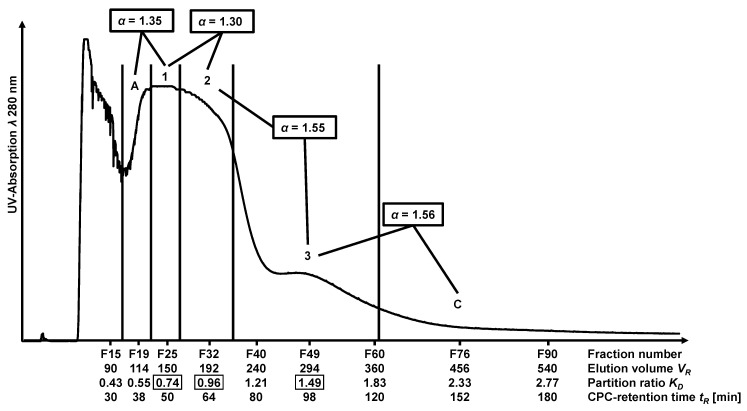
UV chromatogram of the peanut hull extracts FCPC separation at *λ* 280 nm. (**1**), luteolin (*K_D_* = 0.74); (**2**), eriodictyol (*K_D_* = 0.96); (**3**), 5,7-dihydroxychromone (*K_D_* = 1.49); (**A**), fractions 16–22 (*K_D_* = 0.55); (**C**), fractions 62–90 (*K_D_* = 2.33).

For separation by FCPC, the *descending mode* was used, yielding 128 fractions in the *elution mode*. Identical elution order occurred as in the HPCCC experiment (cf. Figure 4). The FCPC fractions were screened by TLC, and fractions were pooled based on the different colorings of the compound spots by anisaldehyde reagent (cf. Supplementary Material, Figure S2). The peak labels A (fractions 16–22, *K_D_* = 0.55) and C (fractions 62–90, *K_D_* = 2.33) in Figure 4 described the sections next to flavonoid-derivate-containing fractions required to calculate separation factor *α*. CPC separation factors were discussed in detail in Section 2.5.

The combined FCPC fractions 23–27 (*K_D_* = 0.74) contained luteolin (**1**) as the main compound (24 mg). Eriodictyol (**2**) was the major compound in fractions 28–36 (*K_D_* = 0.96; 26 mg), and 5,7-dihydroxychromone (**3**) dominated in fractions 37–61 (*K_D_* = 1.49; 28 mg). The final purification step of the combined FCPC fractions containing the three flavonoid derivatives was achieved on an RP-18 semi-preparative column using an isocratic method, thus yielding pure compounds with purities > 98% (*λ* 280 nm).

Separation using FCPC demonstrated the good separation efficiency of countercurrent partition chromatography, as luteolin (**1**) and eriodictyol (**2**) were separated, although solely differing in the double bond position C_2_-C_3_ of the flavonoid A-ring. Luteolin (**1**), eriodictyol (**2**) and 5,7-dihydroxychromone (**3**) could be enriched by CPC separation from a complex peanut hull extract so that subsequent purification by semi-preparative HPLC had to be carried out in the final stage.

### 2.4. Comparison of COSMO-RS and HPLC-UV Based Shake Flask Prediction vs. Experimental HPCCC and FCPC K_D_-Values

In this isolation study for luteolin (**1**), eriodictyol (**2**) and 5,7-dihydroxychromone (**3**), compound-specific *K_D_*-values in the solvent system calculated by *COSMO-RS* and by shake flask experiments, predicted values were compared to the experimental HPCCC and FCPC based *K_D_*-values (cf. Table 3). This process of *K_D_*-comparison required the conversion of the HPCCC and FCPC experimental times into a partition ratio (*K_D_*)-based chromatography scale, as published by Berthod et al. [35]. This approach is used to compare the separation performance of the equipment with different technical designs, such as HPCCC or FCPC (cf. Table 4). The calculation of *K_D_*-values is given in the Supplementary Material Section S5 (Calculation of countercurrent chromatographic separation parameters, cf. Equations (S2)–(S9)) where the experimental HPCCC and FCPC chromatography parameters, such as *retention volumes V_R_*, HPCCC *column volume* and FCPC rotor *column volume V_C_*, *stationary phase volume V_S_*, and the *stationary phase retention values S_F_* were used with the required formulas.

**Figure 5:**
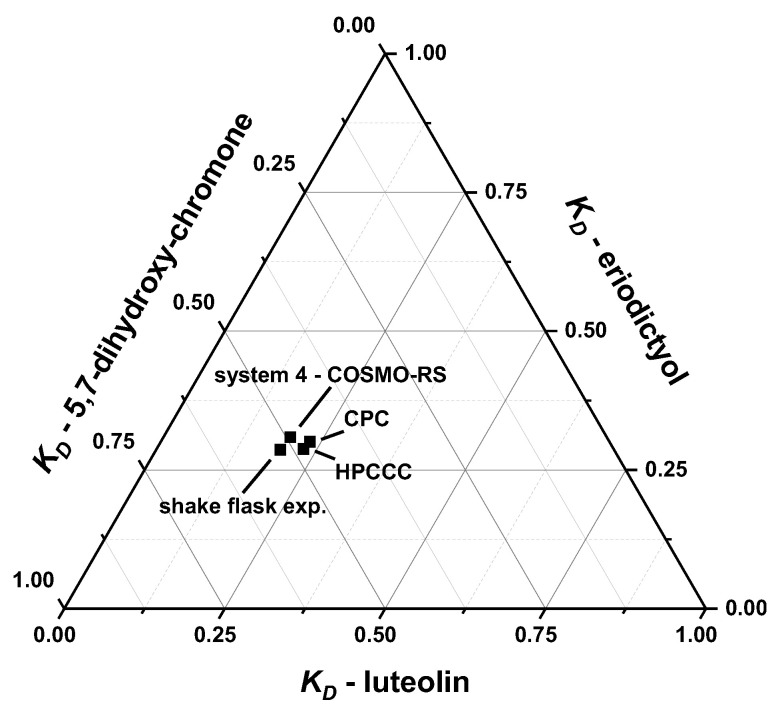
Ternary diagram of *COSMO-RS* calculated and predicted *K_D_*-values by shake flask experimental compared to experimental HPCCC and FCPC *K_D_*-values.

Figure 5 shows a ternary diagram with plotted *K_D_*-values of luteolin (**1**), eriodictyol (**2**) and 5,7-dihydroxychromone (**3**). For this purpose, the *K_D_*-values of the four experiments were normalized and compared. Figure 5 displays that all *K_D_*-values calculated by *COSMO-RS* and those predicted by shake flask experiments were close to the real experimental values of the HPCCC and FCPC separations. Considering the absolute levels, the *K_D_*-values calculated by *COSMO-RS*, also compared to the shake experiments, differ more from the *K_D_*-values determined by HPCCC and FCPC experiments (cf. Table 4).

As additional criteria, *separation factors α* should be considered when selecting an HPCCC and FCPC solvent system. The *α*-values for luteolin/eriodictyol were higher for those calculated with *COSMO-RS* (*α* 1.57) and predicted by shake flask experiments (*α* 1.48) than for the experimental *α*-values with 1.26 for HPCCC as well as 1.30 for FCPC separation. For the pair eriodictyol/5,7-dihydroxychromone the calculated *α*-values using *COSMO-RS* (1.60) were closer to experimental values (HPCCC: *α* 1.68 and FCPC: *α* 1.55) compared to the predicted by shake flaks experiment with *α* 1.81. Both pre-evaluated *α*-values predicted a successful separation of the pairs luteolin/eriodictyol and eriodictyol/5,7-dihydroxychromone, achieved in HPCCC separation. In the FCPC separation, co-elution was present for both metabolite pairs despite the sufficiently large observed *α*-values caused by stronger peak-tailing effects (cf. Table 3).

The prediction and selection of the solvent system based on the calculation by *COSMO-RS* led to a good separation by HPCCC, although the *K_D_*-value of luteolin (**1**) was not in the optimal range (0.4 ≤ *K_D_* ≤ 2.5) [33]. In addition, the calculated *α*-values (based on *COSMO-RS* calculated *K_D_*-values) can be used to estimate whether the pairs under consideration will be sufficiently separated. Overall, the *COSMO-RS* calculations were successfully applied to use an existing solvent system for the separation of the peanut hull extract, in which luteolin (**1**) was separated in addition to eriodictyol (**2**), and 5,7-dihydroxychromone (**3**). Solvent system selection based on the *COSMO-RS* calculations can be applied for both HPCCC and FCPC experiments.

### 2.5. Comparing Chromatographic Performance of HPCCC and FCPC

The chromatographic performance of HPCCC and FCPC for the separation of peanut hull extract was based on the comparison of the determined *separation factors α* and *resolution factors R_S_*. For calculations, the specific HPCCC and FCPC mean $\bar{x}$ *K_D_*-values (cf. Table 4) were used. The classification of the experimental peak ranges for FCPC separation was based on TLC analysis (cf. Supplementary Material, Figure S2). All calculations for relevant countercurrent chromatographic separation parameters were displayed in Supplementary Material (Equations (S2)–(S9)), and results for *α*– and *R_S_*–values for HPCCC/FCPC were given in Table 5, whereby A (fractions 13–15; *K_D_* = 0.41) was used to label the peak maximum before luteolin (**1**). The label B (fractions 24–29; *K_D_* = 0.86) described the peak maximum between eriodictyol (**2**) and 5,7-dihydroxychromone (**3**), whereas C (fractions 37–42; *K_D_* = 1.32) described the section after compound **3**. The *α*– and *R_S_*–values for FCPC were calculated for the pairs shown in Table 5, whereby A described fractions 16–22 (*K_D_* = 0.55) and C fractions 62–90 (*K_D_* = 2.33).

The *α*-value for the pair A/luteolin was 1.32 for HPCCC separation and 1.35 for FCPC (cf. Figure 3 and Figure 4). The *R_S_*-values differed (HPCCC: *R_S_* 1.40 and FCPC *R_S_* 1.20). Therefore no baseline separation (*R_S_* > 1.5) was achieved for the separation of A/luteolin, but it was sufficient to isolate luteolin (**1**) in pure form (cf. TLC analysis, Supplementary Material, Figure S2). In the case of the FCPC separation, however, a complete co-elution for this pair occurred.

The pair luteolin/eriodictyol (HPCCC: *α* 1.26 and FCPC: *α* 1.30) was much better separated by HPCCC (*R_S_* 1.33). In the case of the FCPC run (*R_S_* 1.17), co-elution of luteolin (**1**) and eriodictyol (**2**) was observed.

Only in the case of the HPCCC separation, the *K_D_*-range between 0.77 and 1.03 (label B) separated from eriodictyol (**2**) and 5,7-dihydroxychromone (**3**). For the pair eriodictyol/B, the *α*-value was 1.26, and *R_S_*-value was 1.25. Eriodictyol (**2**) was successfully separated from both luteolin (**1**) and B (cf. TLC analysis, Supplementary Material, Figure S2).

For HPCCC, baseline separation was achieved for the eriodictyol/5,7-dihydroxychromone pair compared to FCPC. The *α*-value for HPCCC separation was 1.68, and the *R_S_*-value was 3.57. By FCPC separation of this pair, the *α*-value was almost comparable (1.55), while the *R_S_*-value was much lower (1.06). Large peak broadening effects in the FCPC influenced the separation result and the calculated *R_S_*.

The pair B/5,7-dihydroxychromone (*α*-value 1.33) was separated by HPCCC, with an *R_S_*-value of 1.67, achieving a complete baseline separation. Co-elution of 5,7-dihydroxy-chromone (**3**) with C is detected by a small *α*-value of 1.16 (*R_S_* 1.25), which was not sufficient to yield a pure compound (cf. Section 2.3). Also, for the FCPC separation, the *α*-value for this pair was 1.56 with an *R_S_*-value of 1.04, and no pure compound could be isolated.

After comparing the *α*-values of the HPCCC and FCPC separations, it was noticed that a separation factor of 1.26 is required for the HPCCC with smaller peak widths to adequately fractionate the flavonoid derivatives in the peanut hull extract. For the FCPC separation, a value of >1.5 should be aspired.

Comparison of the *stationary phase retention S_F_* of the solvent system *n*-hexane/ethyl acetate/methanol/water (1.0/1.0/1.0/1.5; *v*/*v*/*v*/*v*) for the two separation techniques HPCCC (*S_F_* 83%) and FCPC (*S_F_* 91%) indicated that the solvent system is suitable for both of these all-liquid techniques. Very obvious, however, is the superior separation performance of HPCCC versus FCPC for the three flavonoid derivatives (cf. Figure 3 and Figure 4), documented by the respective selectivity and resolution factors (cf. Table 5). HPCCC fractionation for luteolin/eriodictyol and eriodictyol/5,7-dihydroxychromone resulted in well-separated fractions, which could not be achieved by the FCPC device. Despite the observed larger *α*-values for FCPC, the collected fractions showed more co-eluting compounds. The resolution factors *R_S_* of HPCCC separation were higher than those from the FCPC separation, which exhibited very large peak widths. The calculation of the separation factors *α* neglected the real peak width, as only the centers of the peaks will be used in the equation. But for this approach, the *separation factor α* was more important than the *resolution factor R_S_* since it could be calculated directly from *COSMO-RS-based K_D_*-values and thus could also be used as a parameter for solvent system selection by this in-silico method.

## 3. Material and Methods

### 3.1. Chemicals

Double-deionized water (Nanopure^®^, Werner GmbH, Leverkusen, Germany) was used. Ethanol (HPLC grade), methanol (HPLC grade), *n*-hexane (HPLC grade) and acetic acid (LC-MS grade) were purchased from Fisher Scientific (Loughborough, UK). Acetonitrile (HPLC and LC-MS grade) were obtained from Honeywell Speciality Chemicals (Seelze, Germany). Acetic acid (HPLC grade) was purchased from Applichem (Darmstadt, Germany). Ethyl acetate (analytical grade) was purchased from Carl Roth (Karlsruhe, Germany). For TLC, spray reagent anisaldehyde (>98%) from Merck (Darmstadt, Germany) and chloroform (HPLC grade) from VWR Int. S.A.S (Darmstadt, Germany) were used. Dimethyl sulfoxide-d_6_ (99.9% D) from Deutero GmbH (Kastellaun, Germany) and tetramethylsilane (TMS) from Sigma-Aldrich (Deisenhofen, Germany) were used for NMR spectroscopic measurements.

### 3.2. Peanut Hull Preparation and Extraction

Commercial peanuts (roasted in hulls) were peeled, and hulls were separated manually. The hulls were lyophilized (freeze dryer Christ Alpha 2–4, Osterode, Germany) and ground (basic analytical mill A11 IKA, Staufen, Germany) to obtain 58 g of peanut hull powder. A mixture of ethanol and water (80/20; *v*/*v*) was added to the material and was kept for maceration (24 h, under light protection). The extract was filtered and concentrated under a vacuum with a rotary evaporator (40 °C, Laborota 4000, Heidolph Instruments, Schwabach, Germany). This procedure was repeated two times with a fresh ethanol/water mixture. The resulting concentrates were pooled and lyophilized to remove residual aqueous ethanol, to yield 4 g of freeze-dried crude extract of roasted peanut hulls.

### 3.3. Computational Calculation of Phase Equilibrium and K_D_-Values Using COSMO-RS Software

In a preliminary study on the HSCCC separation of the flavonoid-derivatives eriodictyol (**2**) and 5,7-dihydroxychromone (**3**), the solvent system *n*-hexane/ethyl acetate/methanol/water (1.0/1.0/1.0/1.5; *v*/*v*/*v*/*v*) was used [15]. To verify whether luteolin (**1**), eriodictyol (**2**) and 5,7-dihydroxychromone (**3**) could be separated using HPCCC, this and several other solvent systems were pre-evaluated applying the *Conductor-like Screening Model for Real Solvents* (*COSMO-RS*). This model was used to calculate the *partition ratio* (*K_D_*)-values of the three target compounds. Conformers of luteolin (**1**), eriodictyol (**2**) and 5,7-dihydroxychromone (**3**) were calculated in BIOVIA COSMOconfX (Version 22.0.0, Dassault Systèmes, Vélizy-Villacoublay, France) with the *Becke-Perdew functional* (BP) and a *triple-zeta valence polarization with diffuse functions* (TZVPD) and a *fine grid marching tetrahedron cavity* (FINE) template which was used. They indicated a full geometry optimization with the *density functional theory* (DFT) on the BP-TZVP level, with a consecutive BP-def2-TZVPD single-point calculation and a FINE cavity for the *COSMO* calculation. The conformers were considered a Boltzmann-weighted mixture of conformers for the calculations, and the maximum number of conformers was set to 75. Calculated structures were verified to be true minima using vibrational frequency analysis. The LLE compositions of each phase for solvent systems listed in Table 1 were calculated by the *liquid extraction module* for a temperature of 20 °C with the software BIOVIA COSMOthermX (Version 22.0.0, Dassault Systèmes) [24] using the BP_TZVPD_FINE_22.ctd parameterization (cf. Supplementary Material, Table S1). In the following step, the calculated LLEs were used to calculate the *K_D_*-values of luteolin (**1**), eriodictyol (**2**) and 5,7-dihydroxychromone (**3**) for a fixed separation temperature at 28 °C using the *liquid extraction module* of the BIOVIA COSMOthermX software with the BP_TZVP_22.ctd parameterization. *COSMO-RS* allows to calculate the activity coefficient ($\gamma_{i}^{\infty ,s}$) of solute *i* infinitely diluted in solvent *s*, which is used in the following to calculate the *K_D_*-value:

(1) $$\gamma_{i}^{\infty ,s}=exp\left(\left({\;\mu }_{i}^{s}-{\;\mu }_{i}\right)/RT\right)$$

where $\mu_{i}^{s}$ is the chemical potential of the solute *i* in the solvent *s* and $\mu_{i}$ is the chemical potential of pure solute *i*.

The *K_D_*-value is defined as the concentration of solute *i* in the stationary upper phase ($c_{i}^{u}$) divided by the concentration of solute *i* in the mobile lower phase ($c_{i}^{l}$) and can also be expressed as a ratio of the activity coefficients of the solute in infinite dilution ($\gamma_{i}^{\infty }$) multiplied by the molar volumes of the phases (*v*):

(2) $$K_{D}=\frac{c_{i}^{u}}{c_{i}^{l}}=\frac{\gamma_{i}^{\infty ,l}}{\gamma_{i}^{\infty ,u}}\;\times \;\frac{v_{l}}{v_{u}}$$

The phase volume quotient $\frac{v_{l}}{v_{u}}$ was composed of calculated molar volumes by *COSMO-RS* [28,32,36]. The calculated *K_D_*-values of the three flavonoid derivatives in systems 1–10 are shown in Table 6.

### 3.4. HPCCC Separation Procedure

#### 3.4.1. Selection of the Solvent System by Shake-Flask Experiments

To evaluate the *COSMO-RS* calculated *K_D_*-values, classical shake-flask experiments, according to Ito [14], were carried out. 10 mg of peanut hull extract was dissolved in 2 mL of the equilibrated solvent system 4. After 30 s of vigorous shaking, 1 mL of each phase layer was transferred into a new vial and dried under nitrogen gas flow. The residues were re-dissolved in 1 mL of acetonitrile and analyzed by HPLC-UV. The *K_D_*-values were calculated by the measured UV-peak areas at *λ* 280 nm in the upper phase divided by area values obtained for the lower phase.

#### 3.4.2. HPCCC Apparatus and Separations

The flavonoids were separated via *high-performance countercurrent chromatography*—HPCCC (model Spectrum, Dynamic Extractions Ltd., Tredegar, UK). The multilayer coil planet *J*-type HPCCC centrifuge with two self-balanced different-sized columns consisted of two bobbins equipped with one semi-preparative (62.5 mL, 1.6 mm tube i.d.) multi-layer polytetrafluoroethylene coil each. For the HPCCC experiments, both semi-preparative coils were connected in series (125 mL). The temperature was kept constant at 28 °C using a ULK 2002 recirculating chiller from Fryka-Kältetechnik GmbH (Esslingen, Germany). The solvents were pumped with a preparative LC pump K-501 (Knauer Gerätebau GmbH, Berlin, Germany). Elution was monitored at *λ* 280 nm with a Well-Chrom Spectro-Photometer K-2501 detector (Knauer Gerätebau GmbH, Berlin, Germany). Fractions were collected using B-type racks of a Pharmacia Superfrac (Uppsala, Sweden) with a frequency of 2 min/fraction. Eurochrom, 2000 software (Windows version) from Knauer Gerätebau GmbH (Berlin, Germany), was used for chromatographic data acquisition.

Before the chromatographic process, the two-phase solvent system was freshly prepared, and the *n*-hexane/ethyl acetate/methanol/water (1.0/1.0/1.0/1.5; *v*/*v*/*v*/*v*) (HEMWat) mixture was equilibrated in a separatory funnel at ambient temperature. The resulting two-phase layers were separated and shortly degassed by ultra-sonication. The aqueous lower phase was used as the mobile phase, and the organic upper phase as the stationary phase (*head-to-tail* operation mode). The system was filled with the stationary phase, rotation was set to 1590 rpm, and the mobile phase was pumped at a 2.0 mL/min flow rate. After reaching the hydrodynamic equilibrium (mobile phase break-through), the peanut hull extract was dissolved in both solvent phases (1/1; *v*/*v*) at a concentration of 200 mg/mL, filtered over a Chromafil Xtra GF-100/25 fiberglass membrane disc filter (1 µm pore size, 25 mm i.d., Macherey & Nagel, Düren, Germany), and injected over a 5.0 mL sample loop (equiv. to 1000 mg of dry crude extract). The volume of stationary phase displaced during the equilibration procedure (*V_M_*: *mobile phase take-up*) was measured and subtracted from the total coil volume (*V_C_*). The stationary phase content (*V_S_*) remaining in the coil column system represented the *solvent system retention value* (*S_F_*). In the case of HPCCC, *S_F_* was 83%. This value was corrected by the periphery volume *V_ext_* of tubings (cf. Supplementary Material, Section S5). For the chromatographic elution, 200 mL of mobile phase were delivered (equiv. to a *K_D_* of 1.68). After the *elution* mode, the remaining stationary phase was pumped out with nitrogen gas, collected, and separately analyzed. Compounds (**1**) and (**2**) were isolated during the *elution mode* of the *HPCCC* operation and directly used for 1D/2D-NMR analysis (cf. Supplementary Material, Section S6).

### 3.5. FCPC Apparatus and Separation Procedure

The developed HEMWat solvent system successfully operated on the *HPCCC* system and was transferred directly to a hydrostatic system, the *fast-centrifugal partition chromatography* (FCPC^®^-A, KROMATON Sarl, Annoy, France). In this case, the apparatus was operated with a stainless semi-preparative rotor with mounted stainless steel separation disks (rotor column volume with separation ducts: 200 mL). Solvent delivery was done by a preparative LC pump L-6250 Merck-Hitachi intelligent pump (Tokyo, Japan). Elution was monitored at *λ* 280 nm with a Well-Chrom Spectro-Photometer K-2501 detector (Knauer Gerätebau GmbH, Berlin, Germany). Fraction-collecting parameters were identical to the *HPCCC* separation (cf. Section 3.4.2).

The two-phase HEMWat system *n*-hexane/ethyl acetate/methanol/water (1.0/1.0/1.0/1.5; *v*/*v*/*v*/*v*) was separated and degassed by ultra-sonication, and filling of stationary phase to the FCPC rotor column and system was started to rotate at 1000 rpm, while mobile phase delivery (lower phase) was in *descending*-mode at a flow rate of 3.0 mL/min. After reaching the hydrodynamic equilibrium, the dissolved peanut hull extract dissolved in aliquot volumes of the solvent phases (1/1; *v*/*v*) at a concentration of 100 mg/mL was filtered (cf. Section 3.4.2) and injected over a 10 mL sample loop (equiv. 1000 mg crude extract)]. The volume of the displaced stationary phase during hydrodynamic equilibration was measured and subtracted from the total coil volume (cf. Section 3.4.2). Stationary phase retention (*S_F_*) was 91%. During the chromatographic elution procedure, 690 mL of mobile phase was delivered (equiv. to a *K_D_* of 3.55). In analogy to the *HPCCC* experiment, after the *elution mode*, the residual stationary phase was pumped out with nitrogen gas, collected, and analyzed for metabolites.

### 3.6. Analysis of HPCCC Fractions by TLC

The HPCCC and FCPC fractions were analysed by TLC on normal phase silica gel TLC plates (SiO_2_-60 F254, Merck GmbH, Darmstadt, Germany). The plates were developed with the solvent system chloroform/ethyl acetate/methanol/water (25/55/5/1; *v*/*v*/*v*/*v*). Results were visualized by the universal anisaldehyde spray reagent of E. Stahl, consisting of anisaldehyde, concentrated sulphuric acid and glacial acid [37]. Finally, for compound visualization, the plates were heated (105 °C) in a drying oven.

### 3.7. HPLC-UV Analyses

Analysis of the HPCCC and FCPC fractions was made on an HPLC system consisting of an intelligent HPLC pump (PU-280 Plus) with a ternary gradient unit (LG-2080-02) and a 3-line degasser (LG-2080-53). Injections were made by a super-intelligent sampler (AS- 2057 Plus). Peak detection was at *λ* 280 nm with a multiwavelength detector (Md-2010 Plus) and the ChromPass Chromatography Data System Version 1.8.6.1 from Jasco GmbH (Pfungstadt, Germany). HPLC separation was carried out on a C18(2)-column (Aqua 5u, 125 Å, 5 μm, 250 mm × 4.6 mm i.d.) from Phenomenex (Aschaffenburg, Germany) with a guard column of the same material at a flow rate 0.8 mL/min. The mobile phase consisted of 2% aqueous acetic acid (*v*/*v*) (A) and acetonitrile (B) with gradient HPLC conditions for analysis: 0 min (20% B), 20 min (30% B), 40 min (50% B), 50 min (80% B), 55 min (80% B), 60 min (20% B), 70 min (20% B).

### 3.8. Preparative HPLC Separations

The HPCCC fractions containing compound (**3**) were purified by preparative C_18_-HPLC. The solvents were delivered by an HPLC-pump Smartline Pump 1000, with a solvent-controlling system Smartline Manager 5000 and UV-detector K-2600 from Knauer Gerätebau GmbH (Berlin, Germany). A semi-preparative C18-column (Pursuit XRs, 100 Å, 5 µm, 250 mm × 10 mm i.d.) from Agilent Technologies (Waldbronn, Germany) with a guard column of the same material at a flow rate of 2.3 mL/min. The gradient consisted of phase A (water/acetonitrile; 60/40; *v*/*v*) and phase B (acetonitrile), starting with isocratic elution at 0% B for 70 min, followed by a linear gradient up to 100% B in 15 min with 10 min of isocratic elution for column cleaning. Peak detection was done at *λ* 280 nm with the software ChromGate Version 3.1.7 from Knauer Gerätebau GmbH (Berlin, Germany).

### 3.9. HPLC-UV-MS Analyses

For qualification and peak identification purposes, an HPLC-ESI-MS system consisting of a binary HPLC pump (1100 series) and autosampler (1200 series) from Agilent Technologies (Waldbronn, Germany) equipped with an LC-ESI-MS/MS ion-trap system (HCT Ultra ETD II, Bruker Daltonics, Bremen, Germany) was used. Mass spectra were recorded in the negative ionization mode, with the capillary voltage set at 3500 V, end plate −500 V, and capillary exit −115.0 V. Drying gas was nitrogen at 330 °C, and 10.0 L/min flow rate with nebulizer pressure 50 psi, target mass setting *m*/*z* 350, scan range from *m*/*z* 100–2000 in Ultra Scan mode. Compass Hystar Software (Bruker Daltonics) was used for analysis and data collection. HPLC separation was carried out on a C18-column (Aqua 3u, 100 Å, 3 μm, 150 mm × 2.0 mm i.d.) from Phenomenex (Aschaffenburg, Germany) with a guard column of the same material at a flow rate of 0.20 mL/min. The mobile phase consisted of 2% aqueous acetic acid (*v*/*v*) (A) and acetonitrile (B). LC conditions for ESI-MS analysis were 0 min (20% B), 20 min (30% B), 40 min (50% B), 50 min (80% B), 55 min (80% B), 60 min (20% B), 70 min (20% B).

### 3.10. Spectroscopic Measurements

The isolated compounds were identified by the combination of one-dimensional (1D)-NMR (^1^H, ^13^C, DEPT-135) and (2D)-NMR (HSQC, HMBC, COSY) spectroscopic experiments on a Bruker Avance III-HD 500 spectrometer (Bruker Biospin, Ettlingen, Germany) with a probe head at 500.32 (^1^H), and 125.81 (^13^C) MHz, respectively. Samples were recorded in dimethyl sulfoxide-*d*_6_ and referenced to tetramethylsilane (TMS). Chemical shifts *δ* are reported in parts per million [ppm], coupling constants *J* in Hertz [Hz].

## 4. Conclusions

This study presents an efficient method for one-step lab-scale preparation of luteolin (**1**), eriodictyol (**2**) and 5,7-dihydroxychromone (**3**) from the hulls of *A. hypogaea*. Scale-up of this all-liquid chromatographic isolation workflow could access larger amounts of metabolites recovered from an existing production side stream.

As demonstrated, three main flavonoid derivatives of the peanut hull extract were calculated in-silico by *COSMO-RS* software. They resulted in a good approximation of *K_D_*-values to replace laborious shake flask experiments with solvent mixtures. It must be highlighted that both the calculation of the LLE and the *K_D_*-values were carried out in-silico using *COSMO-RS*, and good results were obtained. Thus, it is conceivable to screen many different solvent families in further studies.

Overall, the results had proven a higher separation efficiency and resolution for HPCCC than for FCPC, as documented by chromatographic parameters such as compound selectivity-[*α*] and resolution-[*R*] factors. From aspects of productivity, HPCCC could separate the same amount of crude extract sample in half of the experimental time compared to FCPC. To the best of our knowledge, this is the first report where luteolin (**1**), eriodictyol (**2**) and 5,7-dihydroxychromone (**3**) were isolated from peanut hulls by a single-step HPCCC fractionation.

The three flavonoid derivatives were available in standard quality by this one-step isolation protocol and could be used in bioassays. Due to the good scalability of the CCC technique, this method could subsequently be carried out on a larger scale, e.g., to isolate the bitter masking eriodictyol (**2**) [38].

## Figures and Tables

**Table 1:** Different hexane/ethyl acetate/methanol/water (HEMWat) compositions (*v*/*v*/*v*/*v*) were used for the *COSMO-RS* calculations. * HEMWat solvent system nomenclature [15,26,33,34].

System	[15] *	[26] *	[33] *	[34] *	*n*-Hexane	Ethyl Acetate	Methanol	Water
1		Q	−3		1.5	1.0	1.5	1.0
2		N	0	6	1.0	1.0	1.0	1.0
3			1		4.0	6.0	5.0	5.0
4	1				1.0	1.0	1.0	1.5
5		L	3		1.0	1.5	1.0	1.5
6			4		3.0	7.0	4.0	6.0
7		K			1.0	2.0	1.0	2.0
8		H			1.0	3.0	1.0	3.0
9		G	6		1.0	4.0	1.0	4.0
10		C	7		1.0	9.0	1.0	9.0

**Table 2:** *COSMO-RS* calculated *K_D_*-values and separation factors [*α*] in selected solvent systems.

Solvent Systems Calculated by *COSMO-RS*	*K_lut_*	*K_eri_*	*K_dih_*	*α_lut_*_/*eri*_	*α_eri_*_/_ *_dih_*	*α_dih_*_/_ *_eri_*
1	0.01	0.02	0.13	2.00	6.50	-
2	0.10	0.14	0.38	1.40	2.71	-
3	0.20	0.27	0.53	1.35	1.96	-
4	0.30	0.47	0.75	1.57	1.60	-
5	0.75	1.08	1.18	1.44	1.09	-
6	1.44	1.96	1.61	1.36	-	1.22
7	3.30	4.93	2.78	1.49	-	1.77
8	24	38	9	1.58	-	4.22
9	100	160	21	1.60	-	7.62
10	1610	2597	115	1.61		22.58
shake flask exp. system 4	0.58	0.86	1.56	1.48	1.81	-

Note: shake flask exp.: experimental description cf. Section 3.4.1, use of 10 mg.

**Table 3:** Experimental *K_D_*-values and separation factors from FCPC and HPCCC compared to *COSMO-RS* calculated and shake flask predicted *K_D_*-values.

	*K_lut_*	*K_eri_*	*K_dih_*	*α_lut_*_/_ *_eri_*	*α_eri_*_/_ *_dih_*
FCPC	0.74	0.96	1.49	1.30	1.55
HPCCC	0.54	0.68	1.14	1.26	1.68
system 4—*COSMO-RS*	0.30	0.47	0.75	1.57	1.60
system 4—shake flask exp.	0.58	0.86	1.56	1.48	1.81

**Table 4:** Target compound-specific *K_D_*-values from HPCCC and FCPC.

		HPCCC	Corrected *S_F_*	89%		FCPC	Corrected *S_F_*	96%	
Target Compounds	Solv. System 4 *K_D_*-Calculation (*COSMO-RS*)	Exp. Peak Range Fractions Retention Vol. [mL] Peak Width [mL]	*K_D_* Range *ΔK_D_* Width W	HPCCC Mean Value $\bar{x}$ *K_D_*	*ΔK_D_* = cal. *K_D_* − exp. *K_D_* HPCCC	Exp. Peak Range Fractions Retention Vol. [mL] Peak Width [mL]	*K_D_* Range *ΔK_D_* Width W	FCPC Mean Value $\bar{x}$ *K_D_*	*ΔK_D_* = cal. *K_D_* − exp. *K_D_* FCPC
luteolin (1)	0.30	F16–F19 68–80 12	0.49–0.59 0.10	0.54	−0.24	F23–27 138–162 24	0.68–0.80 0.12	0.74	−0.44
eriodictyol (2)	0.47	F20–F23 84–96 12	0.63–0.74 0.11	0.68	−0.21	F28–F36 168–216 48	0.83–1.08 0.25	0.96	−0.49
5,7-dihydroxy-chromone (3)	0.75	F32–F36 132–148 16	1.06–1.21 0.15	1.14	−0.39	F37–61 222–366 144	1.11–1.86 0.75	1.49	−0.74

**Table 5:** Comparison of values of separation factor *α* and resolutions factor *R_s_* of compound pairs from HPCCC and FCPC.

Fractionated Pairs	HPCCC *α*-Value	HPCCC Resolution Factor *R_s_*	FCPC *α*-Value	FCPC Resolution Factor *R_s_*
A-1	1.32	1.40	1.35	1.20
1-2	1.26	1.33	1.30	1.17
2-B	1.26	1.25	-	-
2-3	1.68	3.57	1.55	1.06
B-3	1.33	1.67	-	-
3-C	1.16	1.25	1.56	1.04

**Table 6:** *COSMO-RS* calculated *K_D_*-values for a separation temperature of 28 °C for luteolin (**1**), eriodictyol (**2**) and 5,7-dihydroxychromone (**3**) in various solvent systems.

System	*K_lut_*	*K_eri_*	*K_dih_*	*α_lut_*_/*eri*_	*α_eri_*_/*dih*_	*α_dih_*_/*eri*_
1	0.01	0.02	0.13	2.00	6.50	-
2	0.10	0.14	0.38	1.40	2.71	-
3	0.20	0.27	0.53	1.35	1.96	-
4	0.30	0.47	0.75	1.57	1.60	-
5	0.75	1.08	1.18	1.44	1.09	-
6	1.44	1.96	1.61	1.36	-	1.22
7	3.30	4.93	2.78	1.49	-	1.77
8	24	38	9	1.58	-	4.22
9	100	160	21	1.60	-	7.62
10	1610	2597	115	1.61	-	22.58

## Data Availability

The data presented in this study are available in Supplementary Material.

## Supplementary Materials

The following supporting information can be downloaded at: https://www.mdpi.com/article/10.3390/molecules28135111/s1, Section S1: All-liquid solvent system prediction for countercurrent chromatography by in-silico calculation by COSMOthermX (Version 22.0.0), Table S1. COSMOthermX phase eqilibrium data at 20 °C with the TZVPD-FINE parametrization; Section S2: HPLC-DAD analysis of the main compound distributions in the phase layers of shake flask experiments by calculation of specific compound partition ratio *K_D_*-values, Table S2. Target compound specific *K_D_*-values from shake flask experiments measured by HPLC-DAD (λ = 280 nm); Section S3: TLC screening of HPCCC fractions, Figure S1. TLC analysis of the HPCCC fractions; (a) fractions 8–24 (b) fractions 25–41; Section S4: TLC screening of FCPC fractions, Figure S2. TLC analysis of the FCPC fractions; (a) fractions 10–30 (b) fractions 31–54; Section S5: Calculation of countercurrent chromatographic separation parameters; Section S6: ESI-MS/MS and 1D/2D-NMR spectral data of isolated compounds, Table S3. ESI-MS/MS data of isolated compounds **1**–**3**, Figure S3. Structure of luteolin **1**, Table S4. NMR data of luteolin **1**, Figure S4. Structure relevant long-range HC-correlation signals observed in the HMBC of luteolin **1**, Figure S5. ^1^H-NMR spectrum of luteolin **1**, Figure S6. ^13^C-NMR spectrum of luteolin **1**, Figure S7. HSQC spectrum of luteolin **1**, Figure S8. HMBC spectrum of luteolin **1**, Figure S9. Structure of eriodictyol **2**, Table S5. NMR data of eriodictyol **2**, Figure S10. Structure relevant long-range HC-correlation signals observed in the HMBC of eriodictyol **2**, Figure S11. ^1^H-NMR spectrum of eriodictyol **2**, Figure S12. ^13^C-NMR spectrum of eriodictyol **2**, Figure S13. HSQC spectrum of eriodictyol **2**, Figure S14. HMBC spectrum of eriodictyol **2**, Figure S15. Structure of 5,7-dihydroxychromone **3**, Table S6. NMR data of 5,7-dihydroxychromone **3**, Figure S16. Structure relevant long-range HC-correlation signals observed in the HMBC of 5,7-dihydroxychromone **3**, Figure S17. ^1^H-NMR spectrum of 5,7-dihydroxychromone **3**, Figure S18. ^13^C-NMR spectrum of 5,7-dihydroxychromone **3**, Figure S19. HSQC spectrum of 5,7-dihydroxychromone **3**, Figure S20. HMBC spectrum of 5,7-dihydroxychromone **3**. References [14,20,35,37] are cited in the supplementary materials.
