# Supplementary material for: A Comparison between High-Performance Countercurrent Chromatography and Fast-Centrifugal Partition Chromatography for a One-Step Isolation of Flavonoids from Peanut Hulls Supported by a Conductor like Screening Model for Real Solvents
Source: Molecules. 2023 Jun 29;28(13):5111. doi: 10.3390/molecules28135111 (PMC10343830; doi:10.3390/molecules28135111)
Supplement: Supplementary file 1 [file molecules-28-05111-s001.zip › molecules-2426244-supplementary.pdf]

<u>**Supplementary Material**</u>

A Comparison between High-Performance Countercurrent Chromatography and Fast-Centrifugal Partition Chromatography for a One-Step Isolation of Flavonoids from Peanut Hulls supported by a Conductor Like Screening Model for Real Solvents

Mats Kiene, Svenja Blum, Gerold Jerz, Peter Winterhalter *

Institute of Food Chemistry, Technische Universität Braunschweig, Schleinitzstrasse 20, 38106 Braunschweig, Germany

* Corresponding author: e-mail address: p.winterhalter@tu-braunschweig.de

Section S1: All-liquid solvent system prediction for countercurrent chromatography by *in-silico* calculation by COSMOthermX (Version 22.0.0)

Table S1. COSMOthermX phase eqilibrium data at 20°C with the TZVPD-FINE parametrization.

HEMWat system	upper phase				lower phase			
	***n*-hexan**	**ethyl acetate**	**methanol**	**water**	***n*-hexan**	**ethyl acetate**	**methanol**	**water**
1	0,6261484	0,2642299	0,09687997	0,012742	0,001761	0,056739	0,367214	0,574286
2	0,4562505	0,3854792	0,12502891	0,033241	6,50E-04	0,046772	0,277996	0,674583
3	0,3260028	0,4481006	0,16517847	0,060718	6,19E-04	0,049112	0,270616	0,679654
4	0,4819859	0,3956727	0,09264124	0,029700	2,69E-04	0,036103	0,211384	0,752244
5	0,3283396	0,4835309	0,1273308	0,060799	2,44E-04	0,038168	0,202029	0,759559
6	0,2197297	0,5279000	0,15522038	0,09715	2,24E-04	0,039463	0,195889	0,764424
7	0,2593752	0,5410544	0,1178212	0,081749	1,29E-04	0,033700	0,158159	0,808012
8	0,1845486	0,6081034	0,09734163	0,110006	5,82E-05	0,029245	0,10998	0,860717
9	0,1439575	0,6469077	0,08145471	0,12768	3,55E-05	0,02708	0,084225	0,88866
10	0,069242	0,7234064	0,04359298	0,163759	1,11E-05	0,023661	0,038771	0,937557

Section S2: HPLC-DAD analysis of the main compound distributions in the phase layers of shake flask experiments by calculation of specific compound partition ratio K_D-values

HPLC-DAD prediction of partition ratio values were calculated with Equation S1 [1]:

K_D-value = peak area A upper phase / peak area A lower phase **Eq. S1**

Table S2. Target compound specific K_D-values from shake flask experiments measured by HPLC-DAD (λ = 280 nm).

compound	retention time [min]	upper phase [mAU*min]	lower phase [mAU*min]	$K_{u/l}$
5,7-dihydroxychromone **3**	12.4	20.3	13.0	1.56
eriodictyol **2**	19.2	108.1	126.1	0.86
luteolin **1**	21.9	54.7	94.3	0.58

Section S3: TLC screening of HPCCC fractions

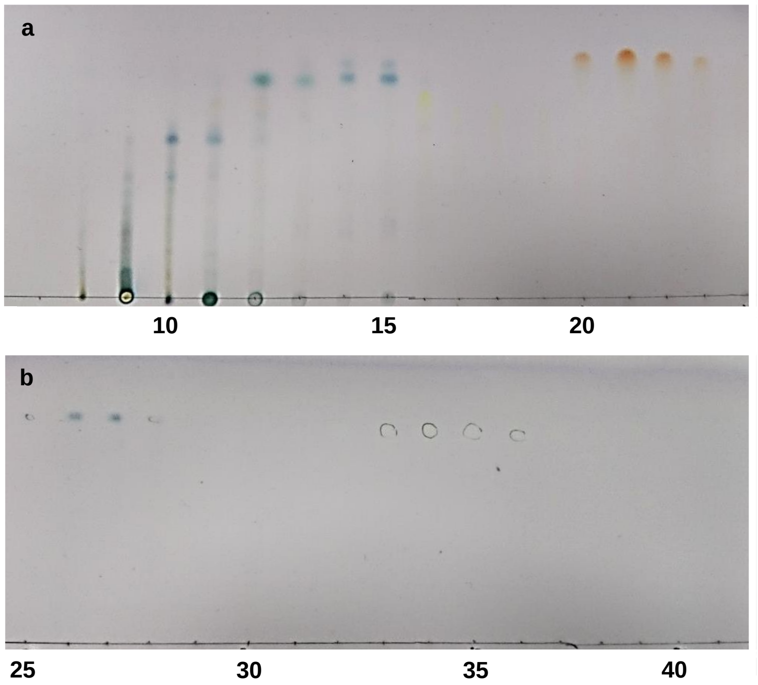

Figure S1. TLC analysis of the HPCCC fractions; a) fractions 8–24 b) fractions 25–41. Normal phase silica gel TLC plates were developed with chloroform/ethyl acetate/methanol/water (25/55/5/1; *v/v/v/v*). Visualization was done using spray reagent anisaldehyde-sulfuric acid-glacial acid (universal reagent Egon Stahl [2]) and thermo-development (105 °C).

Section S4: TLC screening of FCPC fraction

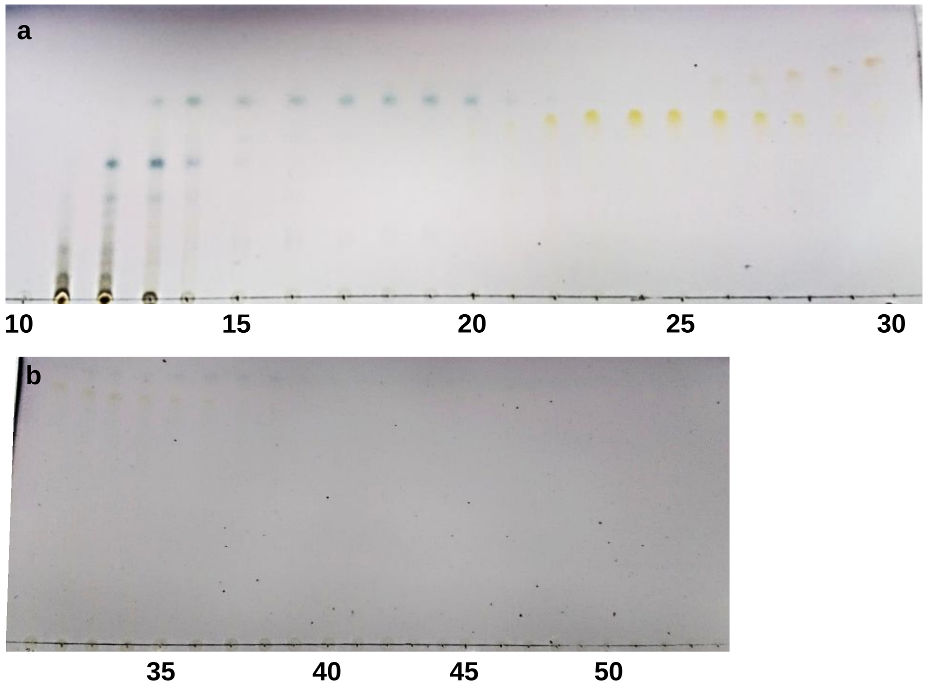

Figure S2. TLC analysis of the FCPC fractions; a) fractions 10–30 b) fractions 31–54. Normal phase silica gel TLC plates were developed with chloroform/ethyl acetate/methanol/water (25/55/5/1; *v/v/v/v*). Visualization was done using spray reagent anisaldehyde-sulfuric acid-glacial acid (universal reagent Egon Stahl [2]) and thermo-development (105 °C). The following fractions were combined in steps of 20, as the contents of the compounds were too low to detect them.

Section S5: Calculation of countercurrent chromatographic separation parameters

The chromatographic elution time was converted over *elution/retention volumes* V_R into their respective *partition ratio values* K_D (cf. Equations S2-S9). The K_D-based projection enables a better comparison between different liquid/liquid chromatography based machine designs such as fast-centrifugal partition chromatography (FCPC), and high-performance countercurrent chromatography (HPCCC). The experimental V_R-values of the three flavonoids luteolin **1**, eriodictyol **2** and 5,7-dihydroxychromone **3** from the FCPC and HPCCC runs were screened by thin-layer chromatography (TLC) and calculated with Equation S2.

Retention volume V_R ***= elution time [min] x flow rate [mL/min]*** **(Eq. S2)**

The S_F*-values* of the used solvent systems were determined by Equations S3a-b using V_C (FCPC: 200 mL; HPCCC: 125 mL), and V_M resulting in the S_F*-value* measured at the hydrodynamic equilibrium.

$V_S = (V_C – V_M)$ **(Eq. S3a)**
$S_F = V_S / V_C$ x 100% **(Eq. S3b)**
FCPC: S_F = 91%
HPCCC: S_F = 83%

V_S: retained experimental stationary phase volume
V_C: column volume/capacity (FCPC: 200 mL; HPCCC: 125 mL)
V_M: volume of mobile phase take up to the coil at equilibrium of FCPC and HPCCC
V_R: retention volume (delivered mobile phase volumes until metabolite elution)
S_F: stationary phase retention in [%]
K_D: partition ratio

The determined S_F*-value* in the experiments is corrected by the *extra column volume* V_{ext} (FCPC: 10 mL; HPCCC: 7 mL) [3] of the connecting periphery tubing in the FCPC and HPCCC set-ups, using Equations S4-S6

Corrected $V_M = V_M - V_{ext}$ **(Eq. S4)**
FCPC*: corr.* V_M = 18 mL - 10 mL = 8 mL
HPCCC*: corr.* V_M = 21 mL - 7 mL = 14 mL

Corrected $V_S = V_C$ **– corrected** V_M **(Eq. S5)**
FCPC*: corr.* V_S = 200 mL - 8 mL = 192 mL
HPCCC*: corr.* V_S = 125 mL - 14 mL = 111 mL

Corrected S_F **= corrected** V_S / V_C **(Eq. S6)**
FCPC*: corr.* S_F = 192 mL / 200 mL x 100% = 96%
HPCCC*: corr.* S_F = 111 mL / 125 mL x 100% = 89%

It should be noted that in general a high S_F*-value* directly correlate to a higher resolution and efficiency of the FCPC and HPCCC separations.

The compound and solvent system specific partition ratio K_D-values in the FCPC and HPCCC runs were calculated by the Equation S7.

During *elution-mode*: [4]

$$K_D = (V_R - \text{corrected } V_M) / \text{corrected } V_S \qquad \text{(Eq. S7)}$$

The *separation factor α* and *resolution factor* R_S depend on the distances between peaks and the peak widths that will be compared. The calculation of both factors depend on the determined K_D-values.

$$\alpha = K_{D2} / K_{D1} \text{ (with } K_{D2} > K_{D1}) \qquad \text{(Eq. S8)}$$

The calculation of the *resolution factor* R_S described how well two peaks are separated at the baseline from each other.

$$R_S = 2\,(K_{D2} - K_{D1}) / (W_2 + W_1) \qquad \text{(Eq. S9)}$$

W_n: peak width at baseline

Section S6: ESI-MS/MS and 1D/2D-NMR spectral data of isolated compounds

Table S3. ESI-MS/MS data of isolated compounds **1-3**.

comp.	*MW [g/mol]*	*ESI polarity*	*pseudo molecular ion*	*parent ion m/z*	*m/z from MS²*
1	286	neg.	$[M-H]^-$	285	257(37), 241(68), 223(16), 217(35), 199(100), 175(29), 151(19), 149(12), 133(11)
2	288	neg.	$[M-H]^-$	287	269(3), 151(100), 135(10), 125(1), 107(7)
3	178	neg.	$[M-H]^-$	177	133(100)

[a] Base peaks are underlined, MS² relative intensities in braclkets

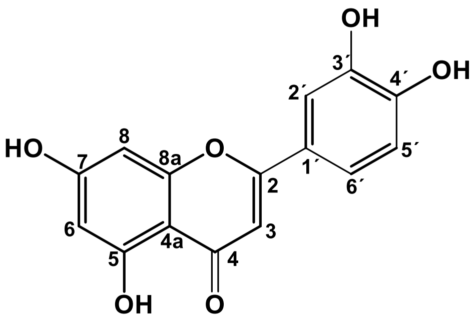

Figure S3. Structure of luteolin **1**.

Table S4. NMR data of luteolin **1**.[a]

**Position**[b]	**^{13}C: δ_C [ppm] (DEPT135)**	**^{1}H: δ_H [ppm]; mult., *J* [Hz]**[c]	**HMBC**
2	163.9 (C)		
3	102.8 (C)	6.68; s	C-2, 4, 4a, 1’
4	181.6 (C)		
4a	103.6 (C)		
5	161.4 (C)		
6	98.8 (CH)	6.20; d, *J* = 2.1	C-4a, 5, 7, 8
7	164.1 (C)		
8	93.8 (CH)	6.45; d, *J* = 2.1	C-4a, 6, 7, 8a
8a	157.6 (C)		
1’	121.4 (C)		
2’	113.3 (CH)	7.40; d, *J* = 2.1	C-2, 3‘, 4‘, 6‘
3’	145.7 (C)		
4’	149.9 (C)		
5’	116.0 (CH)	6.90; d, *J* = 8.3	C-1’, 3’, 4’
6’	119.0 (CH)	7.42; dd, J_1 = 8.3/ J_2 = 2.3	C-2, 2’, 4’
5-OH	-	12.98; s	C-4a, 5, 6

[a] Solvent: dimethylsulfoxide-d_6; tetramethylsilane δ 0.00 ppm for ^{1}H, δ 39.5 ppm for ^{13}C; ^{1}H observed frequency 500.32 MHz.
[b] For numbering of the carbon atoms, see the formula, assignment of C-H via HSQC data.

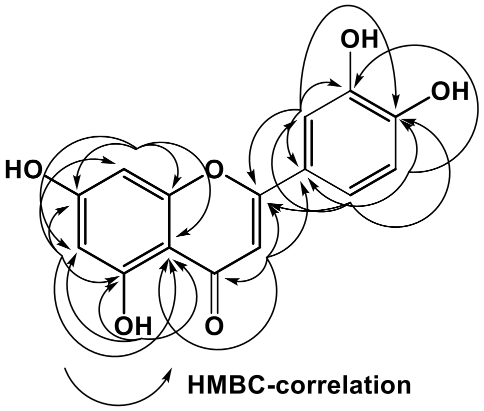

Figure S4. Structure relevant long-range HC-correlation signals observed in the HMBC of luteolin **1**.

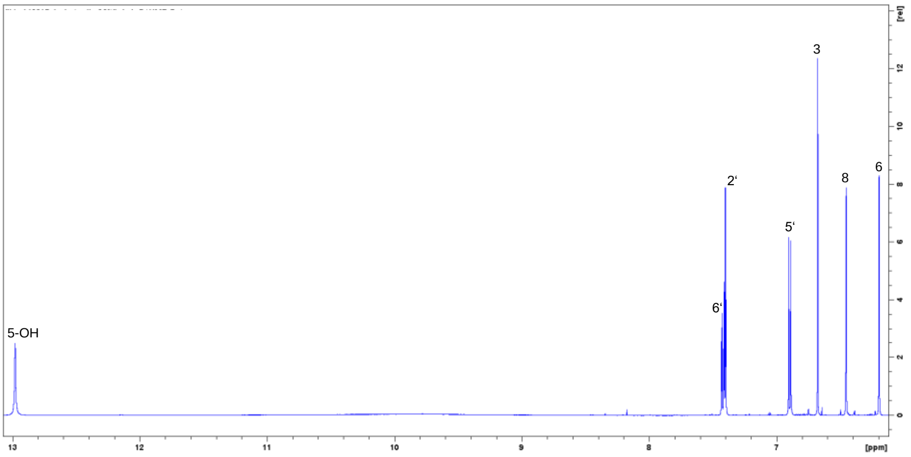

Figure S5. ^{1}H-NMR spectrum of luteolin **1**.

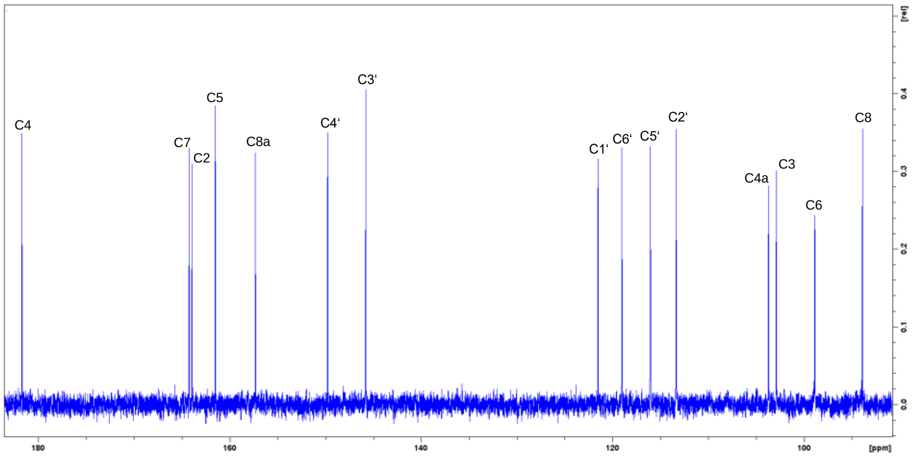

Figure S6. ^{13}C-NMR spectrum of luteolin **1**.

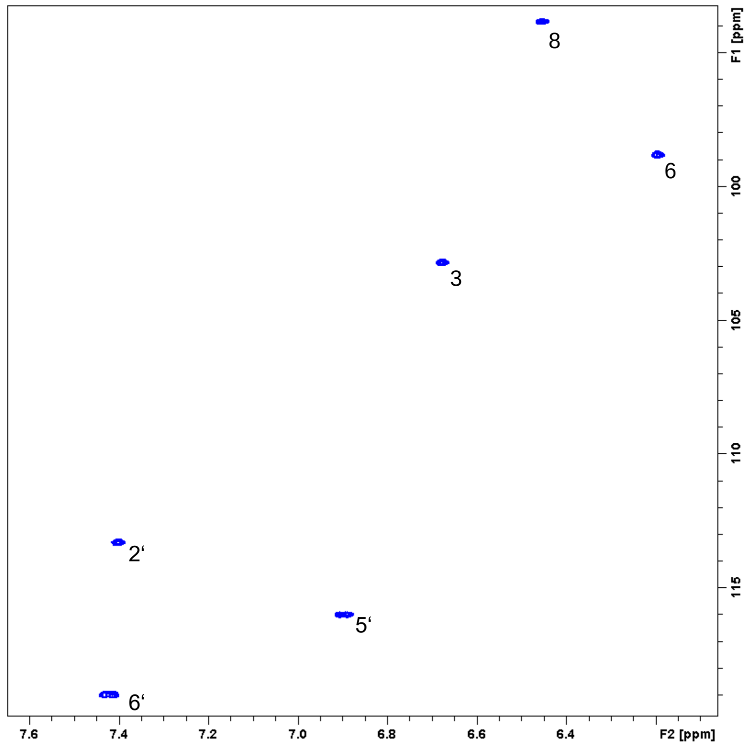

Figure S7. HSQC spectrum of luteolin **1**.

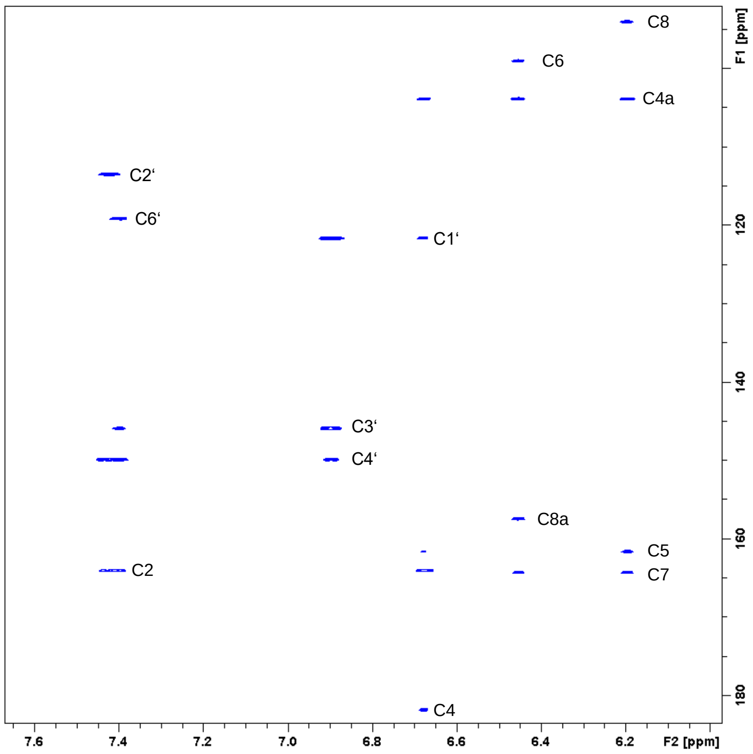

Figure S8. HMBC spectrum of luteolin **1**.

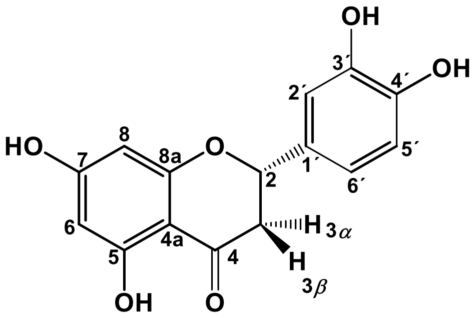

Figure S9. Structure of eriodictyol **2**.

Table S5. NMR data of eriodictyol **2**.[a]

Position[b]	^{13}C: δ_C [ppm] (DEPT135)	^{1}H: δ_H [ppm]; mult., J [Hz][c]	HMBC
2	78.4 (CH)	5.38; dd, J_1 = 12.5/ J_2 = 3.1	C-3, 4, 8a, 1', 2', 6'
3	42.0 (CH_2)	α 3.19; dd, J_1 = 17.1/ J_2 = 12.5	C-2, 4, 1'
		β 2.67; dd, J_1 = 17.1/ J_2 = 3.2	C-4, 4a, 1'
4	196.3 (CH_q)		
4a	101.7 (C)		
5	163.4 (C)		
6	95.6 (CH)	5.87; d, J = 2.1	C-4a, 5, 7, 8
7	166.5 (C)		
8	94.9 (CH)	5.88; d, J = 2.1	C-4a, 6, 7, 8a
8a	162.8 (C)		
1'	129.3 (C)		
2'	114.2 (CH)	6.87; s	C-2, 1', 3', 4', 6'
3'	145.1 (C)		
4'	145.6 (C)		
5'	115.2 (CH)	6.74; m	C-1', 3', 4'
6'	117.9 (CH)	6.74; m	C-2, 1' ,2', 4'
5-OH	-	12.14; s	C-4a, 5, 6

[a] Solvent: dimethylsulfoxide-d_6; tetramethylsilane δ 0.00 ppm for ^{1}H, δ 39.5 ppm for ^{13}C; ^{1}H observed frequency 500.32 MHz.
[b] For numbering of the carbon atoms, see the formula, assignment of C-H via HSQC data.
[c] For CH_2 groups with diastereotopic protons α and β indicate the deshielded and shielded nucleus, respectively.

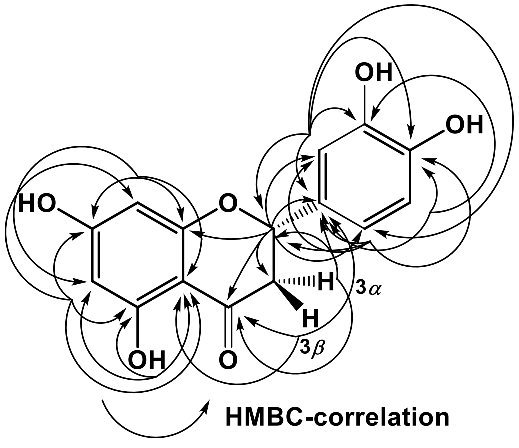

Figure S10. Structure relevant long-range HC-correlation signals observed in the HMBC of eriodictyol **2**.

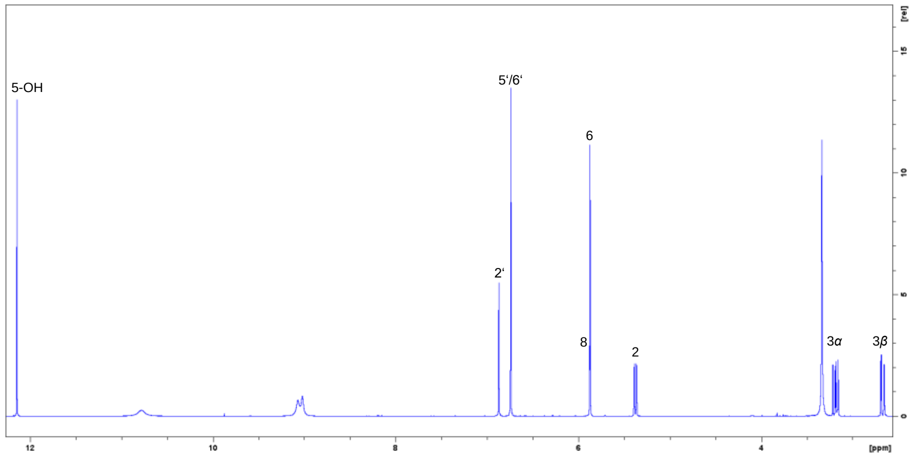

Figure S11. ^{1}H-NMR spectrum of eriodictyol **2**.

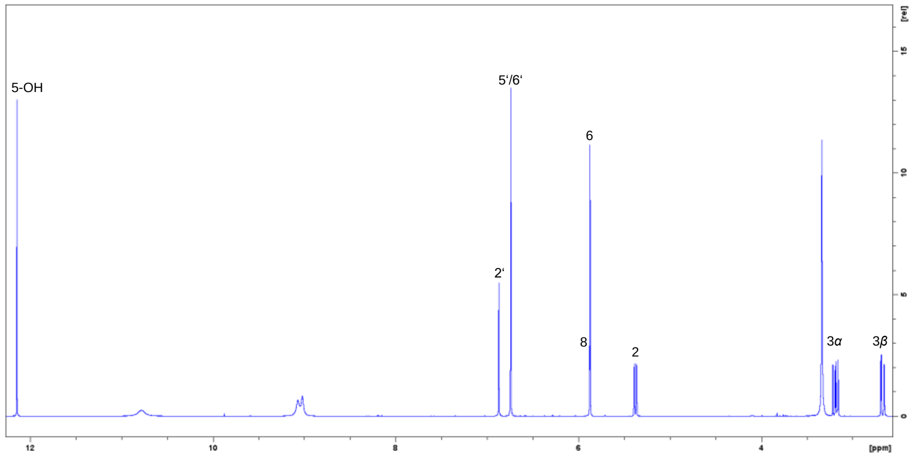

Figure S12. ^{13}C-NMR spectrum of eriodictyol **2**.

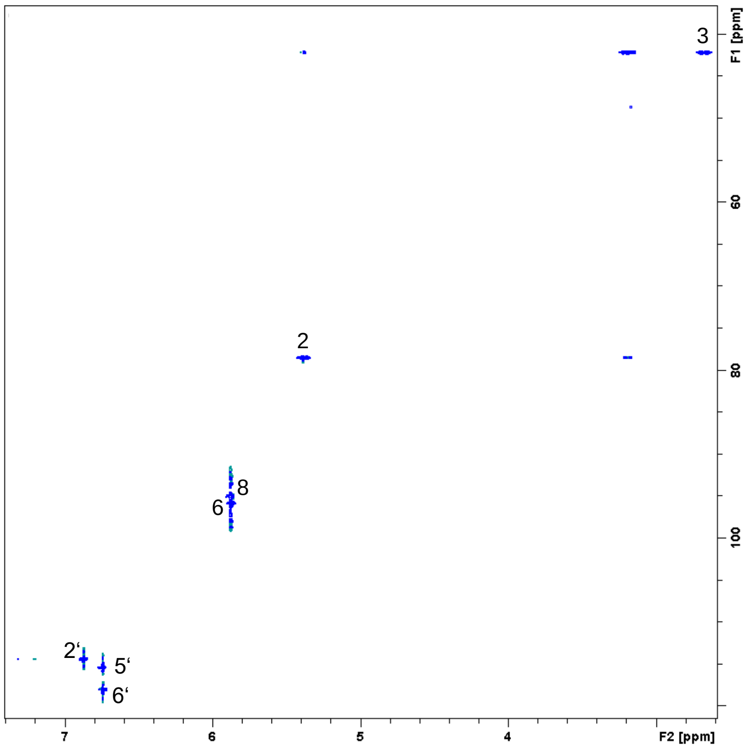

Figure S13. HSQC spectrum of eriodictyol **2**.

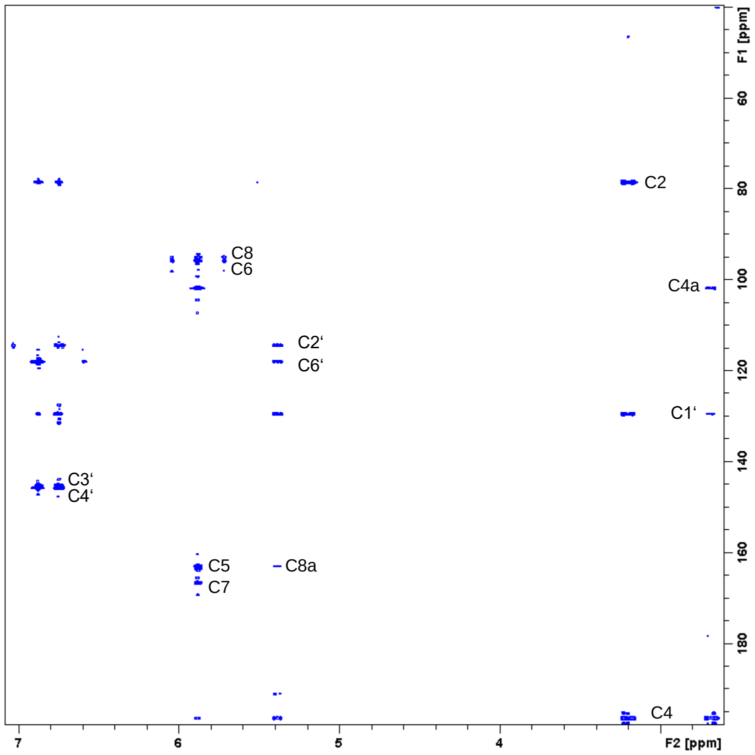

Figure S14. HMBC spectrum of eriodictyol **2**.

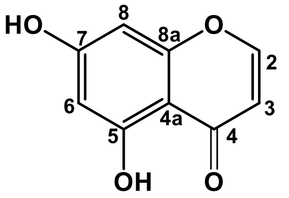

Figure S15. Structure of 5,7-dihydroxychromone **3**.

Table S6. NMR data of 5,7-dihydroxychromone **3**.[a]

Position[b]	^{13}C: δ_C [ppm] (DEPT135)	^{1}H: δ_H [ppm]; mult., J [Hz][c]	HMBC
2	157.2 (CH)	8.16; d, J = 5.9	C-3, 4, 8a
3	110.3 (CH)	6.26; d, J = 5.9	C-2, 4a
4	181.0 (C)		
4a	104.5 (C)		
5	161.5 (C)		
6	99.1 (CH)	6.16; d, J = 2.0	C-4a, 5, 7, 8
7	165.0 (C)		
8	94.0 (CH)	6.33; d, J = 2.0	C-4a, 6, 7, 8a
8a	157.7 (C)		
5-OH	-	12.69; s	C-4a, 5, 6

[a] Solvent: dimethylsulfoxide-d_6; tetramethylsilane δ 0.00 ppm for ^{1}H, δ 39.5 ppm for ^{13}C; ^{1}H observed frequency 500.32 MHz.
[b] For numbering of the carbon atoms, see the formula, assignment of C-H via HSQC data.

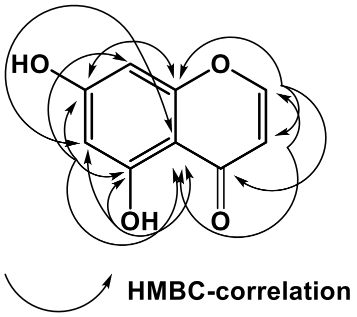

Figure S16. Structure relevant long-range HC-correlation signals observed in the HMBC of 5,7-dihydroxychromone **3**.

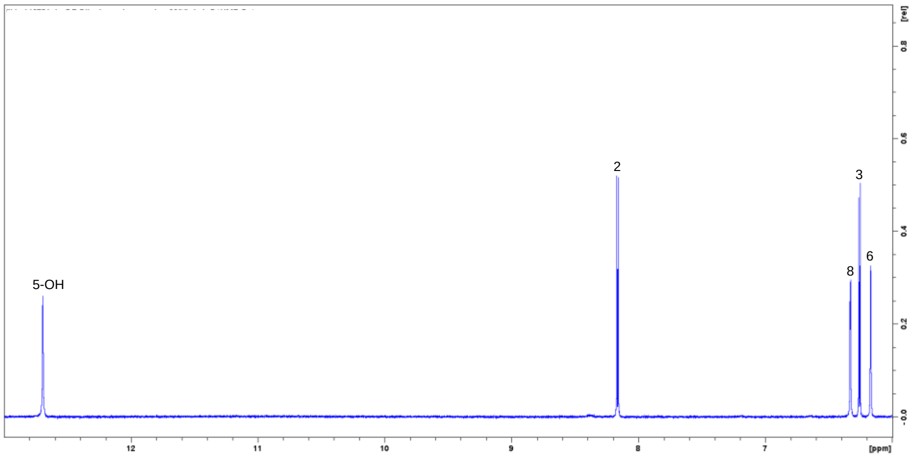

Figure S17. ^{1}H-NMR spectrum of 5,7-dihydroxychromone **3**.

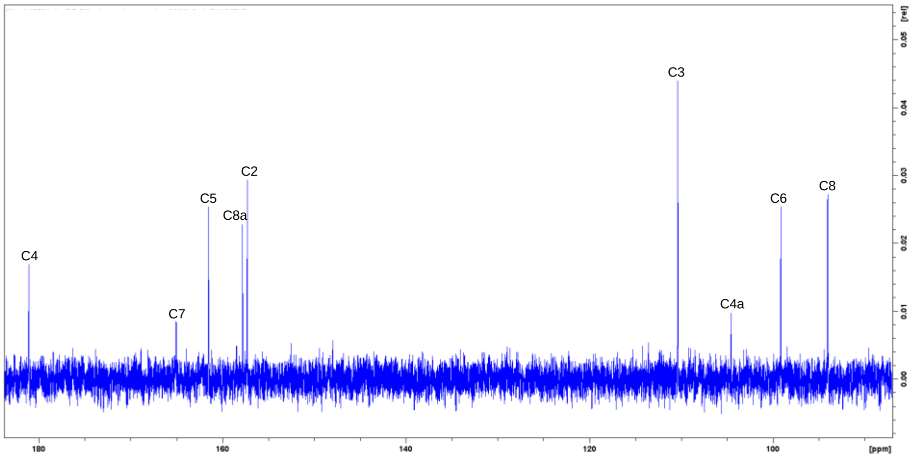

Figure S18. ^{13}C-NMR spectrum of 5,7-dihydroxychromone **3**.

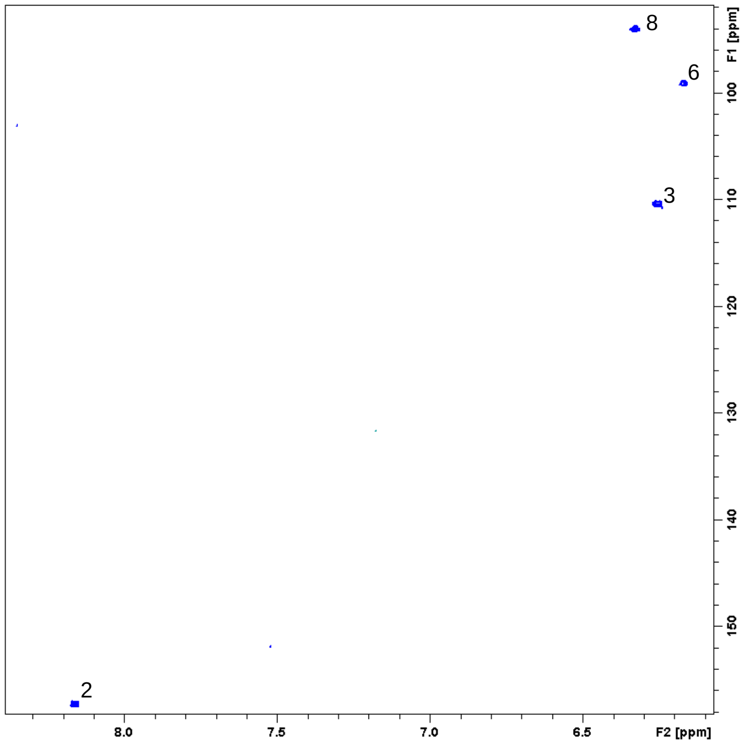

Figure S19. HSQC spectrum of 5,7-dihydroxychromone **3**.

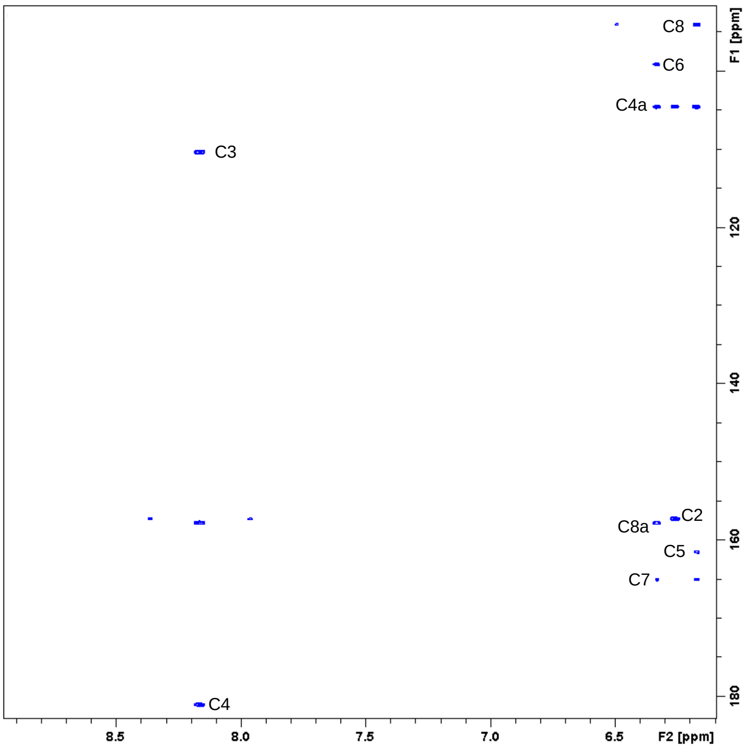

Figure S20. HMBC spectrum of 5,7-dihydroxychromone **3**.

References

1. Ito, Y. Golden Rules and Pitfalls in Selecting Optimum Conditions for High-Speed Counter-Current Chromatography. *J. Chromatogr. A* **2005**, *1065* (2), 145–168.

2. Stahl, E.; Kaltenbach, U. Dünnschicht-Chromatographie: VI. Mitteilung. Spurenanalyse von Zuckergemischen Auf Kieselgur G-Schichten. *J. Chromatogr. A* **1961**, *5* (1961), 351–355.

3. Costa, F. das N.; Vieira, M.N.; Garrard, I.; Hewitson, P.; Jerz, G.; Leitão, G.G.; Ignatova, S. *Schinus terebinthifolius* Countercurrent Chromatography (Part II): Intra-Apparatus Scale-up and Inter-Apparatus Method Transfer. *J. Chromatogr. A* **2016**, *1466*, 76–83.

4. Berthod, A.; Friesen, J.B.; Inui, T.; Pauli, G.F. Elution−Extrusion Countercurrent Chromatography: Theory and Concepts in Metabolic Analysis. *Anal. Chem.* **2007**, *79* (9), 3371–3382.
